# Bayesian Causal Methods for Environmental Accountability Studies with Heterogeneous Effects

**DOI:** 10.1007/s40572-026-00555-5

**Published:** 2026-09-17

**Authors:** Dafne Zorzetto, Emma Landry, Michele Guindani, Donatello Telesca, Valeria Edefonti, Roberta De Vito, Falco J. Bargagli-Stoffi

**Affiliations:** 1 Department of Biostatistics, Brown University, Providence, RI 02912 USA; 2 Department of Biostatistics, University of California Los Angeles, Los Angeles, CA 90095 USA; 3 Department of Clinical Sciences and Community Health - Dipartimento di Eccellenza 2023-2027, University of Milan, Milan, Italy; 4 Fondazione IRCCS Ca’ Granda, Ospedale Maggiore Policlinico, Milan, Italy; 5 Department of Statistics, Sapienza University of Rome, Rome, Italy

**Keywords:** Environmental accountability, Bayesian nonparametrics, Causal inference, Heterogeneous treatment effects

## Abstract

**Purpose of Review:**

Environmental accountability studies evaluate whether regulatory interventions achieve their intended benefits. These studies fall into two broad categories: *indirect accountability*, which estimates the effects of exposure to higher levels of air pollution and uses them to indirectly predict policy impacts, and *direct accountability*, which evaluates specific interventions directly. The purpose of this review is to evaluate recent advances in causal inference and flexible Bayesian statistical modeling to support environmental accountability studies.

**Recent Findings:**

Recent studies, in both direct and indirect environmental accountability, deeply rely on causal inference to produce robust inferences and provide relevant and actionable insights to policy-makers and practitioners. In this context, heterogeneous treatment effects are central to both approaches as it is critically important to understand which subpopulations are most vulnerable (or resilient), requiring methods that can characterize effect variation. Bayesian nonparametric (BNP) methods have been proposed for this task due to their flexibility and ability to cluster units into coherent subgroups.

**Summary:**

We present a selection of BNP methods designed or adapted for each type of accountability, focusing on complementary approaches based on Bayesian Additive Regression Trees (BART) and Dependent Dirichlet Processes (DDP). Through Monte Carlo simulations, we compare these methods, identify their respective advantages, and provide recommendations for adoption and implementation in environmental accountability research.

## Introduction: Causal Inference to Inform The Accountability Framework in Environmental Health

The Health Effects Institute introduced the “environmental accountability” framework to systematically evaluate the health effect of exposure to air pollution and whether environmental regulations, aimed at reducing such exposure, can achieve their intended public health goals [1]. This framework recognizes that demonstrating health benefits from air quality improvements requires moving beyond associational studies toward rigorous causal evaluation of specific interventions [2, 3]. The accountability framework links regulatory actions to changes in emissions, ambient concentrations, exposures, and ultimately health outcomes where each link requires distinct methodological approaches [3]. Landmark cohort studies established that long-term fine particulate matter (PM$_{2.5}$) exposure increases cardiovascular and respiratory mortality [4, 5], results subsequently confirmed using administrative data covering tens of millions of older Americans [6]. These foundational findings, together with evidence of health effects at concentrations below current regulatory standards [7], underscore the need for methods that can rigorously quantify both the magnitude of exposure-response relationships and evaluate the effectiveness of policies designed to reduce exposures.

Zigler and Dominici [3], building on the framework laid out by the Health Effect Institute [1], distinguished two distinct approaches to environmental accountability: indirect and direct accountability. *Indirect accountability* answers: “What is the relationship between pollution exposure and health?” These studies estimate the effects of exposure to higher levels of air pollution (often dichotomized at a policy relevant threshold, as we will see below) and use them to predict policy impacts, assuming that the estimated relationship would hold under regulatory implementation. *Direct accountability* answers: “Did this specific environmental regulatory intervention actually impact health?” Under the causal inference framework, invoked by Zigler and Dominici [3], these studies have the potential to evaluate well-defined policies (e.g., nonattainment designations, scrubber installations) and whether they caused health changes. The distinction is fundamental: indirect studies inform what *might* happen under hypothetical interventions, whereas direct studies reveal what *did* happen following actual policy implementation. Evidence from both approaches is needed to provide a complementary and well-rounded assessment of exposure–health relationships and the effectiveness of actions to reduce exposures, ultimately guiding protection and mitigation strategies [3, 8].

Both approaches must address heterogeneous treatment effects (HTE), which are ubiquitous in environmental health due to differential susceptibility, exposure patterns, and policy implementation [2, 9–11]. Substantial evidence indicates that air pollution health effects vary by age, with the elderly showing heightened vulnerability [11, 12]; by socioeconomic status and race/ethnicity [6]; and by pre-existing health conditions including diabetes, heart failure, and chronic lung disease [13]. These susceptibility patterns have direct implications for environmental health equity: communities that bear disproportionate pollution burdens are often those with elevated vulnerability to its effects [14]. In indirect accountability, key heterogeneous effects questions include who is most vulnerable to pollution exposure and where interventions will have greatest impact. In direct accountability, the focus shifts to where the intervention worked (and, potentially, through what mechanisms). Traditional approaches based on pre-specified subgroups are limited: they may miss important heterogeneity along unanticipated dimensions and suffer from multiple testing issues [15, 16]. Data-adaptive methods that discover heterogeneous groups while maintaining principled inference are therefore needed.

Bayesian nonparametric (BNP) methods offer a data-adaptive solution by placing priors over flexible function spaces, allowing the data to reveal heterogeneity patterns while borrowing strength across similar observations [17–19]. This review focuses on four BNP approaches that provide flexible, data-adaptive solutions to HTE estimation in environmental health: Bayesian Causal Forests with post-hoc clustering (BCF+CART) and the Confounder-Dependent Bayesian Mixture Model (CDBMM) for indirect accountability [20, 21]; the Confounders-Aware SHared atoms BAyesian mixture (CASBAH) and Bayesian Principal Causal Forests (BPCF) for direct accountability [22, 23].

We want to emphasize that among the landscape of BNP methods for causal inference, we focus on these four approaches because they represent, to our knowledge, the current set of BNP methods purpose-built at the intersection of four defining criteria: (i) BNP modeling of the outcome-exposure relationship, (ii) heterogeneous treatment effect estimation and subgroup discovery, (iii) seamless integration of the accountability framework for environmental health regulation evaluation, and (iv) code availability, ensuring reproducibility and facilitating adoption by the broader environmental research community. This distinguishes them from BNP methods applied to environmental health that target exposure-response curves or average causal effects [24–26] or methods that do not provide a fully reproducible code. Furthermore, we want to note that, given these four defining criteria, this work should be approached as selective review rather than a broad review of Bayesian causal methods in environmental health.

The four selected methods further span two conceptually distinct dimensions that are central to environmental health policy evaluation: the accountability pathway (indirect versus direct) and the mechanism for heterogeneity discovery (tree-based partitioning versus mixture-model clustering), providing a comprehensive view of current methodological options within a coherent organizational structure. Furthermore, we note that, while alternative frequentist methods are also available [16, 27], the reviewed Bayesian causal inference methods share key features attractive for environmental health: they provide full posterior inference for uncertainty quantification, they provide a flexible modeling of the relationship between exposure to higher levels of air pollution and health outcomes, and enable heterogeneity discovery without requiring pre-specification [28, 29].

These methods are evaluated through extensive Monte Carlo simulations under controlled conditions for both indirect and direct accountability studies. In causal inference settings, researchers typically do not have access to the ground truth (that is, the causal effect for each unit in the study). Simulation studies are therefore essential for assessing how the methods respond to different definitions and structures of treatment effect heterogeneity under scenarios with controlled ground truth. In these settings, model performance can be compared vis-a-vis with the data-generating-process ground truth to provide an evaluation of the methods performance [30]. In both the indirect and direct accountability simulation studies, we compare two different specifications of heterogeneous causal effects: (i) modeling them as continuous functions of covariates or (ii) assuming that units belong to latent groups defined by covariates. The former corresponds to Scenario 1 in both simulation studies, whereas the latter corresponds to Scenario 2. This comparison allows us to assess which modeling approaches are better suited to each data generating process and how effectively they address the corresponding causal research questions.

The remainder of the paper is organized as follows. Section 2 presents the causal inference framework, distinguishing between indirect and direct accountability with their respective estimands and assumptions. Sections 3 and 4 detail the four methods , highlighting their respective strengths and the settings in which each is most appropriate. In these Sections, we also evaluate their performance through Monte Carlo simulations. Section 5 provides practical guidance, a discussion of the approaches, and outlines future directions.

## Causal Inference in Environmental Health: Estimands, Assumptions and Causal Identification

### Setup and Notation

We adopt the potential outcomes framework for causal inference [31]. For unit *i*, let $Y_i$ denote the observed outcome. For each unit *i*, we postulate the existence of two potential outcomes, $Y_i(1)$ and $Y_i(0)$, representing the potential outcomes under treatment and control, respectively. Let $X_i$ denote a vector of pre-treatment covariates. In environmental health accountability studies, units may be individuals, geographic areas (e.g., ZIP codes, counties), or time periods, depending on the study design. Under the standard assumption of stable unit treatment value [32], the observed outcome is $Y_i = T_i Y_i(1) + (1-T_i) Y_i(0)$, where $T_i \in \{0,1\}$ indicates treatment assignment. In the following sections, we introduce the causal question that direct and indirect accountability seek to answer, along with the underlying causal assumptions and the corresponding identification results. We review these components because, although not novel, they form the backbone of any applied causal analysis: without estimands, assumptions, and identification, causal methods cannot deliver causal estimates.

We note that binary treatment often requires dichotomization of a continuous exposure [16, 33]. While this reduces the richness of the exposure, proper causal estimands can still be defined, as argued by Lee et al [34]. These authors also emphasize that binarization is particularly valuable when the binarization threshold corresponds to real-world policy cutoffs (such as in the case of National Ambient Air Quality Standards, NAAQS), making the estimand directly actionable for regulatory decisions (which is the ultimate desiderata of accountability studies). In fact, under this framework, treatment does not presuppose an implemented intervention: rather, it denotes a well-defined hypothetical contrast between exposure regimes. In the following, aligning with the literature, we refer to such treatment as *exposure*.

Continuous exposure frameworks also exist [35] and have been applied in environmental epidemiology [36] but none of these approaches provides a data-driven characterization of the heterogeneity in the exposure effect without having to pre-specify ex ante the subgroups of analysis (on the limitations of pre-specified HTE analysis we refer the reader to Athey and Imbens [15]). Furthermore, we note that a full discussion of Bayesian causal inference framework is outside the scope of this paper. We refer the reader interested in a topical discussion of this framework to the excellent reviews proposed by Oganisian and Roy [37], Li et al. [38], and Linero and Antonelli [39].

### Indirect Accountability

As introduced above, indirect accountability studies how to answer the question: *What is the causal relationship between pollution exposure above a certain threshold and health?* These studies estimate the effect of exposure to higher level of air pollution on health outcomes, for example, the effect of PM$_{2.5}$ on mortality. These estimates can be used to *indirectly* predict the health impacts of proposed or implemented policies especially when using, as dichotomization thresholds, policy relevant values such as the NAAQS. The underlying assumption, in this case, is that the estimated causal relationship would persist under regulatory implementation.

#### Causal Estimands

Under the indirect accountability framework, we can define several causal estimands, i.e., causal quantities related to the research question of interest. We start from the *average treatment effect* (ATE) which is defined as

$$\begin{aligned} \tau _{\text {ATE}} = \mathbb {E}[Y_i(1) - Y_i(0)], \end{aligned}$$

representing the expected causal effect of exposure across the entire population. In this setting, the ATE might represent the average effect of high versus low PM$_{2.5}$ exposure on mortality risk. As argued above, high and low exposures can be defined based on policy-relevant thresholds such as NAAQS as proposed by Lee et al. [16, 33].

When exposure effects vary across subpopulations, the *conditional average treatment effect* (CATE) captures this heterogeneity:

$$\begin{aligned} \tau (x) = \mathbb {E}[Y_i(1) - Y_i(0) \mid X_i = x]. \end{aligned}$$

The CATE is central to indirect accountability because it reveals *who is most vulnerable* to higher pollution exposures and, indirectly, *where interventions might have the greatest impact*. For instance, the effect of higher PM$_{2.5}$ exposures may differ by age, socioeconomic status, baseline health, or geographic region.

A coarser summary is the *group average treatment effect* (GATE) [21]:

1 $$\begin{aligned} \tau _g = \mathbb {E}[Y_i(1) - Y_i(0) \mid G_i = g], \end{aligned}$$

where $G_i$ denotes group membership. When groups correspond to covariate regions $\mathcal {R}_g \subseteq \mathcal {X}$, the GATE reduces to the CATE averaged over that region: $\tau _g = \mathbb {E}[\tau (X_i) \mid X_i \in \mathcal {R}_g]$. Groups may be pre-specified based on policy-relevant categories (e.g., race, age, urbanicity) or discovered from data using data-adaptive methods reviewed in this paper. It is worth noting that traditional approaches relying on pre-specified subgroups, extensively explored in the literature [6, 36], are limited: they may miss important effect heterogeneity and suffer from multiple testing problems [15]. The BNP methods reviewed below provide data-adaptive discovery of heterogeneous groups while maintaining principled inference.

It is useful to clarify at the outset how three notions invoked throughout this review—heterogeneous effect *estimation*, subgroup *discovery*, and *clustering*—relate, as they are distinct but nested goals rather than competing ones. *Estimation* concerns quantifying the effect itself, whether at the individual level through the CATE *τ (x)* or at the group level through the GATE $\tau _g$ [40, 41]. The CATE is the most granular characterization of heterogeneity, describing how the effect varies across the full covariate space $\mathcal {X}$; the GATE is a coarsening that averages *τ (x)* over a region $\mathcal {R}_g$, yielding a small number of interpretable, policy-relevant summaries [40, 41]. *Discovery* concerns determining the partition $\{\mathcal {R}_g\}$ itself—that is,*where the boundaries between groups are drawn*—from the data, rather than fixing it ex ante [16, 42]. *Clustering* is one data-adaptive mechanism by which discovery can be carried out, and is the route taken by the BNP mixture methods reviewed below [21]; tree-based methods instead recover the partition directly through recursive splitting of $\mathcal {X}$ [15, 16]. In all cases, the GATEs are the substantive target—they answer *who* is most vulnerable to higher exposures—while the partition is the means of reporting them at an actionable granularity.

#### Identifying Assumptions

Causal identification in the context of indirect accountability relies on three core assumptions which are standard in the causal inference literature in observational studies [43]: stable unit treatment value assumption (SUTVA), unconfoundedness, and positivity.

First, *SUTVA* requires no interference between units and no hidden versions of exposure [32]. We note that this assumption is needed to properly define the potential outcomes. In environmental accountability, SUTVA may be violated when pollution from one area affects neighboring areas (spatial spillovers) or when individuals migrate between high- and low-exposure regions. Dichotomizing a continuous exposure might violate the no hidden versions of the exposure component of the assumption. Accountability studies should therefore carefully define the unit of analysis and exposure contrast to minimize these concerns [34]. Bayesian methods to account for spatial spillovers have been proposed [44–47], but are outside the scope of this review.

Second, *unconfoundedness* requires exposure assignment to be independent of potential outcomes conditional on observed covariates: $(Y_i(1), Y_i(0)) \perp \!\!\!\perp T_i \mid X_i$. This assumption, often called “no unmeasured confounding,” is central to observational studies [43]. In environmental accountability research in air pollution, key confounders include socioeconomic status, access to healthcare, baseline health conditions, meteorological factors, and co-pollutants. The plausibility of ignorability depends critically on the richness of available covariates; when violations are suspected, sensitivity analysis can assess robustness [48–52].

Third, *positivity* requires that all units have a non-zero probability of receiving each exposure level: $0< P(T_i = 1 \mid X_i = x) < 1$ for all *x* in the support of *X*. Violations occur when certain covariate combinations deterministically predict exposure, for example, if all urban areas exceed a pollution threshold while all rural areas fall below it. Positivity violations restrict the subpopulations for which causal effects can be estimated and may necessitate focusing inference on regions of covariate overlap [53, 54]. Together, unconfoundedness and positivity are referred to as *strong ignorability*.

#### Identification

Under SUTVA and strong ignorability, causal estimands can be expressed in terms of observable quantities, that is, they can be *identified* from the observed data. For the ATE:

$$\begin{aligned} \tau _{\text {ATE}} = \mathbb {E}_X\bigg [\mathbb {E}[Y_i \mid T_i = 1, X_i] - \mathbb {E}[Y_i \mid T_i = 0, X_i]\bigg ], \end{aligned}$$

where the inner expectations are conditional means of the observed outcome, estimable from data. Similarly, the CATE (and GATE) is identified as:

$$\begin{aligned} \tau (x) = \mathbb {E}[Y_i \mid T_i = 1, X_i = x] - \mathbb {E}[Y_i \mid T_i = 0, X_i = x]. \end{aligned}$$

The BNP methods reviewed below (i.e., BCF+CART and CDBMM) estimate these conditional expectations flexibly while providing full posterior uncertainty quantification and data-adaptive discovery of effect heterogeneity.

### Direct Accountability

Direct accountability studies answer: *Did this specific environmental regulatory intervention actually impact health?* Rather than estimating general exposure-response relationships, these studies evaluate well-defined policy interventions (henceforth, referred to simply as *intervention*), such as nonattainment designations under the Clean Air Act or scrubber installation requirements [55–59], and whether they caused measurable health changes. Whereas indirect studies inform what *might* happen under a proposed policy, direct studies reveal what *did* happen following implementation of a particular policy.

A fundamental challenge in direct accountability is that environmental regulations do not uniformly affect pollution levels across all areas. Comparing health outcomes between regulated and unregulated areas therefore conflates the effect of the regulation with heterogeneity in compliance or implementation. Principal stratification addresses this challenge by asking: *Where did the intervention work? Through what mechanisms?* We note that mediation analysis offers a complementary perspective: principal stratification targets heterogeneity in policy compliance or implementation, while mediation analysis decomposes effects into direct and indirect pathways [60–64]. Due to space constraints, this review focuses on principal stratification; for mediation approaches in environmental health, see, e.g., [63, 65, 66].

#### Direct Accountability Through Principal Stratification

*Principal stratification* provides a framework for causal inference when a post-intervention (often referred to as *post-treatment*) intermediate variable $P_i$ lies between the intervention and outcome [67]. Each unit belongs to a principal stratum defined by $S_i = (P_i(0), P_i(1))$, the joint potential values of the intermediate under each intervention condition. Because principal strata are defined by potential outcomes under both intervention conditions, they are latent but conceptually represent an inherent characteristic of units that does not depend on intervention assignment [68]. The principal causal effect within stratum *s* is:

2 $$\begin{aligned} \tau (s) = \mathbb {E}[Y_i(1) - Y_i(0) \mid S_i = s]. \end{aligned}$$

As an example, we illustrate principal stratification using the 2005 revision of the NAAQS [22, 69]. This revision designated counties exceeding PM$_{2.5}$ thresholds as “non-attainment,” requiring them to develop State Implementation Plans to reduce pollution. Here, intervention $T_i$ is non-attainment designation, the intermediate $P_i$ is the subsequent change in PM$_{2.5}$ concentration, and the outcome $Y_i$ is the change in mortality. Principal strata are defined by how each county’s pollution responds to the designation. The *associative negative stratum* comprises counties for which non-attainment designation reduced PM$_{2.5}$, i.e., these are “compliers” whose pollution levels responded to the regulatory pressure. The *dissociative stratum* comprises counties where pollution was unaffected by the designation, either because they were already improving or because implementation was ineffective. The *associative positive stratum* comprises counties for which PM$_{2.5}$ increased despite designation, potentially reflecting broader economic trends, behavioral responses, or measurement issues.

Estimating principal causal effects separately within each stratum answers distinct policy questions. The associative negative effect reveals whether health benefits accrue to areas that achieved actual pollution reductions. The dissociative effect assesses whether the regulation affects health through pathways other than pollution reduction, for instance, through increased health monitoring or behavioral changes in designated areas.

#### Identifying Assumptions

Because $S_i$ involves potential values of the intermediate under both intervention conditions, principal strata are never directly observed. Identification therefore requires assumptions beyond those used for indirect accountability [52, 70]. Below we review the most common assumption in the principal stratification literature, even though, as discussed in Schwartz et al. and, more recently, in Zorzetto et al. [22, 71] some of these assumptions (e.g., monotonicity) can be relaxed under a Bayesian modeling of the latent principal strata.

*Principal ignorability* requires that intervention assignment is independent of potential outcomes conditional on both observed covariates and principal stratum membership. In the NAAQS context, this implies that among counties belonging to the same stratum (e.g., those where pollution would decrease regardless of designation), the actual designation is effectively random conditional on baseline characteristics.

*Monotonicity* assumes that no unit responds to intervention in the “wrong” direction. For NAAQS, monotonicity requires that no county experiences pollution reductions *only* when not designated as non-attainment. This assumption rules out “defiers” and simplifies the stratum structure. Monotonicity can be plausible when regulatory mechanism operates in a consistent direction, but may fail if compensating behaviors occur [68, 72].

#### Identification

Under SUTVA, strong ignorability, and the principal-specific assumptions above, principal causal effects can be expressed as functions of observable quantities. For stratum *s*, the principal effect is identified as [22, 73]:

3 $$\begin{aligned} \tau (s) = \int _{\mathcal {X}} \bigg[ \mathbb {E}[Y_i \mid T_i = 1, X_i = x, S_i = s] \\ - \mathbb {E}[Y_i \mid T_i = 0, X_i = x, S_i = s]\bigg] \, dF_X(x \mid S_i = s), \end{aligned}$$

where the conditioning on $S_i = s$ is operationalized through models for $P_i(0)$ and $P_i(1)$ given covariates. The BNP methods reviewed below—CASBAH and BPCF—estimate these quantities by jointly modeling the outcome and intermediate variable distributions, imputing missing potential outcomes, and propagating uncertainty about stratum membership through posterior inference.

### The Critical Role of Assumptions in Environmental Health Studies

The causal interpretations above rest entirely on the identifying assumptions. No statistical method, Bayesian or frequentist, parametric or nonparametric, can verify whether these assumptions hold in a given application. SUTVA cannot be tested from observed data because interference patterns involve counterfactual comparisons across intervention assignments of other units. Unconfoundedness cannot be confirmed because unmeasured confounders are, by definition, unobserved. Positivity violations can sometimes be diagnosed empirically, but near-violations may be difficult to detect. Principal ignorability and monotonicity involve latent strata that are never directly observed.

When these assumptions fail, causal identification fails, regardless of how flexible or sophisticated the estimation method may be. Bayesian nonparametric methods offer important advantages, i.e., flexible modeling of complex relationships, honest uncertainty quantification, and principled handling of missing potential outcomes, but they cannot remedy fundamental identification problems. Researchers must therefore justify assumptions using substantive knowledge, employ sensitivity analyses to assess robustness to violations [52, 74], and interpret results with appropriate caution. This is especially true in the context of environmental health studies where some of these assumptions, as discussed in the sections above, might need expert knowledge to be justified.

Thus, we want to stress that the methods reviewed in the following sections should be understood as tools for estimation and inference *given* that identification holds, not as substitutes for careful causal reasoning.

## Indirect Accountability: Evaluating Effects of Exposures

As discussed in the previous sections, indirect accountability studies assess how health outcomes are affected by higher pollution exposures. Characterizing heterogeneity in this context is essential for identifying vulnerable populations, uncovering key socioeconomic and demographic factors that underlie these disparities, and designing more effective and targeted policy interventions and mitigation strategies.

The complex relationships among the variables involved, together with the likely presence of heterogeneous effects, motivate the use of flexible modeling strategies such as BNPs.

Well-known BNP approaches include models that employ the Dirichlet process prior [75], along with its extensions to dependent Dirichlet processes (DDP) [76, 77], and Bayesian Additive Regression Trees (BART) [78]. Their use in causal inference has grown substantially in recent years [39], due to several desirable features, including: (i) the ability to capture complex response distributions through flexible model specifications, (ii) the capacity to naturally handle missing data within a probabilistic framework, (iii) straightforward posterior sampling for uncertainty quantification of estimates, and (iv) the possibility to incorporate prior knowledge about the phenomenon under study.

We highlight that under the Bayesian paradigm, statistical inference targets the posterior distribution, obtained from combining the data likelihood with the prior distributions that encode beliefs surrounding the unknown model quantities. For the DPP and BART-based models that we consider, posterior distributions are analytically intractable. Therefore, samples are obtained through simulation-based algorithms, specifically Markov Chain Monte Carlo (MCMC). Inference is then direct using the obtained posterior samples. Estimation of model parameters, such as means or variance; as well as derived quantities of interest, including population-, group-, or unit-level causal estimands, is achieved by evaluating these functionals at each MCMC iteration. Point estimates are typically obtained by considering the posterior mean or median, whereas uncertainty quantification relies on credible intervals built from empirical quantiles of the given samples.

In this section, we compare two Bayesian causal inference models. The first is the Bayesian Causal Forest (BCF), introduced by Hahn et al. [20], which leverages the BART prior. The second is the Confounder-Dependent Bayesian Mixture Model (CDBMM) proposed by Zorzetto et al. [21], which is based on a probit stick-breaking process [79], a specific construction within the broader class of DDPs. Other important contributions to BNP in causal inference include Hill (2011) [17], who helped introduce BART to this community, as well as subsequent work extending DDP-based approaches to causal estimands [18, 80].

### BCF+CART: Two-Stage Approach

The key innovation of BCF [20] is the definition of a novel nonlinear regression model that combines two BART priors and incorporates the propensity score in the modeling of the outcome of interest. This design targets settings with small effect sizes and strong confounding by observed covariates, improving regularization and interpretability of heterogeneous exposure effect estimates.

BCF follows the S-learner specification [38], i.e. modeling both exposure groups jointly via a Single regression function:

4 $$\begin{aligned} Y_i \mid \textbf{x}_i, T_i =t_i \sim \mu (\textbf{x}_i, \hat{\pi }(\textbf{x}_i)) + \tau (\textbf{x}_i)\cdot t_i + \epsilon _i; \end{aligned}$$

where $\mu (\cdot )$ and $\tau (\cdot )$ are assigned independent BART priors [78], and the error term $\epsilon _i$ follows a standard Gaussian distribution. Here, $\hat{\pi }(\textbf{x}_i)$ is an estimate of the propensity score [81] $\pi (\textbf{x}_i)=\text {Pr}(T_i = 1 \mid \textbf{x}_i)$. Incorporating the propensity score, when the outcome model is specified using an S-learner, helps attenuate the dependence between confounders and exposure, promotes balance in the distribution of observed covariates across exposure groups, and ultimately supports more robust causal inference [82, 83].

In Eq. 4, $\mu (\cdot )$ captures the prognostic contribution of confounders, while $\tau (\cdot )$ directly models the exposure effect as a covariate-dependent quantity, accommodating potential effect heterogeneity, as defined as follows:

$$\begin{aligned}\begin{gathered} \mu (\textbf{x}_i, \hat{\pi }_i(\textbf{x}_i)) = \mathbb {E}[Y_i \mid T_i = 0, \textbf{x}_i, \hat{\pi }_i(\textbf{x}_i)],\\ \tau (\textbf{x}_i) = \mathbb {E}[Y_i \mid T_i = 1, \textbf{x}_i] - \mathbb {E}[Y_i \mid T_i = 0, \textbf{x}_i, \hat{\pi }_i(\textbf{x}_i)]. \end{gathered}\end{aligned}$$

BART priors facilitate the detection of complex interactions and local discontinuities among the covariates, allows for invariance to monotone transformations of the predictors, and typically requires minimal parameter tuning [20, 78]. Specifically, the BART prior represents an unknown function as a sum of many piecewise regression trees. Each tree consists of a set of internal decision nodes, which define a partition of the covariate space, and a set of terminal nodes/leaves corresponding to each element, each associated with a parameter value.

While the estimated $\hat{\tau }(\textbf{x}_i)$ provides unit-level heterogeneity, interpretation becomes challenging as the number of confounders grows. In this setting, estimating the GATE (see Eq. 1) can help identify subgroups that are particularly vulnerable or resistant. Subsequently, a classification and regression trees (CART) [84] can be applied to the estimated $\hat{\tau }(\textbf{x}_i)$ to summarize and interpret the results of the BCF model, identifying groups of units that share similar exposure effects within each group while differing across groups. Specifically, CART partitions the units into non-overlapping groups by similarities of the values of the variables, through binary tree structures. The prior probability of a split in the tree at depth *d* is equal to $\alpha \Delta (1+d)^{-\beta }$, where *α * controls the base propensity to split and *β * controls how fast that rate decays with depth. Given the tree structure, observations assigned to the same terminal node (leaf) defines a cluster with similar covariate profiles.

This BCF+CART approach allows us to capture uncertainty at the levels of individual exposure effect and heterogeneous exposure effect, although quantifying uncertainty at the group level is not straightforward. This procedure is often referred to as a *fit-the-fit* approach and was introduced in the seminal BCF paper by Hahn et al. [20] and further extended in recent contributions [40, 72].

### CDBMM: Native Group Discovery via Mixtures

The CDBMM exploits a key property of mixture models: their ability to induce data-driven partitions of units based on both potential outcomes and confounders. Specifically, it places a DDP prior [76, 77] on the conditional distribution of the outcome given the exposure level, following a T-learner specification [38], i.e. modeling each group separately with Two functions:

$$\begin{aligned} Y_i \mid \textbf{x}_i, t \sim \sum _{l \ge 1} \omega _{l}^{(t)}(\textbf{x}_i) \mathcal {N} (\eta ^{(t)}_{l},\sigma ^{2(t)}_{l}), \quad \forall t \in \{0,1\}. \end{aligned}$$

The marginal outcome distributions for the two exposure levels are modeled as independent Gaussian mixtures, while confounders enter through the weight sequences $(\omega _{l}^{(\cdot )}(\textbf{x}_i))_{l \ge 1}$. These weights (i) govern heterogeneity in the response distributions across units, (ii) induce dependence between the two exposure-specific marginal distributions, which is leveraged for the imputation of missing potential outcomes, and (iii) determine the marginal partitions of the units induced by the mixture model. Specifically, the weights follow the probit stick-breaking process [79] definition:

$$\begin{aligned}\begin{gathered} \omega _{l}^{(t)}(\textbf{x}_i) = V_{l}^{(t)}(\textbf{x}_i) \prod _{r<l}\left\{ 1 - V_{r}^{(t)}(\textbf{x}_i)\right\} ,\\ V_{l}^{(t)}(\textbf{x}_i) = \Phi \left( \alpha _{l}^{(t)}(\textbf{x}_i)\right) , \quad \alpha _{l}^{(t)}(\textbf{x}_i) \sim \mathcal {N}\left( \beta _{0l}^{(t)} + \textbf{x}_i^T \beta _{l}^{(t)}, 1 \right) , \quad \beta _{l}^{(t)} {\mathop {\sim }\limits ^{\textrm{iid}}} \mathcal {N}(0, 20). \end{gathered}\end{aligned}$$

where the sequence $(V^{(t)}_l)_{l\ge 1}$ is an independent stochastic process that takes values in [0, 1], and the function $\Phi (\cdot )$ indicates the probit function. The prior for the parameters in the Gaussian kernel are defined as follows

5 $$\begin{aligned} \eta _l^{(t)} {\mathop {\sim }\limits ^{iid}} Norm(0,20), \text{ and } \sigma _l^{(t)} {\mathop {\sim }\limits ^{iid}} \text{ InvGamma }(5,1), \end{aligned}$$

where the hyperparameters are set to the default values used in the Gibbs sampling algorithm of the HTEBayes package. The hyperparameter for *η * specifies a weakly informative prior, suggesting a average causal effect centered at zero and a relatively diffuse distribution. In contrast, the hyperparameters for *σ * are chosen to concentrate the prior distribution near zero, thereby inducing a priori small variability within each cluster. When prior knowledge is available, these hyperparameters can be modified accordingly.

The T-learner estimates separate outcome models for the treated and control groups, thereby providing greater flexibility to capture complex outcome surfaces and heterogeneous treatment effects. In contrast, the S-learner relies on a single predictive model that incorporates the treatment indicator as a covariate, often resulting in more stable estimates, particularly when treatment effects are small. As a result, the S-learner may be preferable in settings with limited sample sizes, whereas the T-learner is generally better suited for scenarios characterized by substantial treatment-effect heterogeneity.

CDBMM allows to directly estimate the partition space of the groups to estimate the GATE (see Eq. 1) and their point estimation. The partition space is obtained using the Cartesian product of the cluster partition of the two marginal models, where the point estimation is estimated with the solution proposed by Wade and Ghahramani [85] based on the decision theory that minimizes the posterior expectation of a loss function using Binder’s loss [86] or Variation of Information [87]. In CDBMM, the final partition can be interpreted through the Cartesian product of the clustering induced by the two exposure-specific mixture models.

A terminological clarification is in order, as the BNP-mixture machinery distinguishes two partitions. Consistently with the BNP literature, we use *cluster* for the sets that partition the observations within a single exposure level $t \in \{0,1\}$, obtained as a byproduct of the infinite-mixture specification through the procedure of Wade and Ghahramani [85]. These arm-specific clusters are then combined across *t* to form the *groups*: the final partition for which distinct GATEs are computed. The distinction is not merely notational. Clusters are an intermediate, arm-specific device internal to the model; groups are the effect-relevant objects that carry the substantive interpretation. Two units may fall in the same cluster under *t=1* yet different clusters under *t=0*, and it is precisely this combination—not the within-arm clustering itself—that defines their shared or distinct causal effect [21].

### Simulation Study

To assess the operating characteristics of these two competing methods under controlled conditions, we conduct an extensive simulation study. The data generation processes are constructed following the simulated scenario proposed in the two papers [20, 21], respectively: Scenario 1 mimics the BCF construction in the case of heterogeneous causal effect and non-linear functions, and Scenario 2 is scenario 5 in Zorzetto et al. [21] where the true number of clusters is known and equal to 5. Both Scenarios impose nonlinear data generating processes for the outcome and a set of confounding variables that simultaneously affect both exposure and outcome. See Table 1 for details. Both models are straightforwardly estimated in R software environment, through the publicly available implementations of the algorithms. Specifically, the bcf package, available on GitHub, is used for estimating BCF; rpart is employed for CART-based analyses; and the HTEBayes package, also available on GitHub, is used to estimate the CDBMM. The functions are used with the default settings of parameters. In particular, rpart uses the 10-fold cross-validation, where the tree is estimated by fitting it on 9 folds of the data, and its predictive performance is evaluated on the remaining fold to select the optimal tree size. Given the final pruned tree, each observation is mapped to a leaf/node.

**Table 1:** Parameters and distributions for the simulation scenarios under indirect accountability

	Scenario 1	Scenario 2
Source	Heterogeneous and nonlinear case in Hahn et al. (2020)[20]	Scenario 5 in Zorzetto et al. (2024)[21]
Covariates	$X_{1:3} \sim \mathcal {N}(0,1)$, $X_4 \sim \text {Be}(0.5)$	$X_j \sim \text {Be}(\pi _j) \; j=1,\dots , 5$
	$X_5 \sim \text {Categorical}(1,2,3)$	*π = (0.4,0.6,0.3,0.5,0.2) *
Exposure	$T\sim \text {Be}(f_1(X_1,X_3,X_5))$	$T\sim \text {Be}(f_2(X_{1:5}))$
	$f_1 = 0.8 \Phi (3 \mu _x / sd(\mu _x)) - 0.5 X_1) + 0.05$	$f_2 = \text {logit}(0.4X_1+0.6X_2-0.3X_3+0.2X_4 X_5)$
Clusters	No cluster structure	$$i \in {\left\{ \begin{array}{ll} 1 & \text {if } X_{i1}=1, X_{i2}=1; \\ 2 & \text {if } X_{i1}=0, X_{i3}=1; \\ 3 & \text {if } X_{i1}=0, X_{i3}=0, X_{i4}=1; \\ 4 & \text {if } X_{i1}=0, X_{i3}=0, X_{i4}=0; \\ 5& \text {otherwise}. \end{array}\right. }$$
Outcome	$Y = \mu _x + T(1 + 2X_2 X_4) + \epsilon$	$Y_i(t) \sim \sum _{c=1}^5 I_{(i \in c)}\mathcal {N}(\eta _{t,c}, 0.2)$
	$\mu _x = -6 +g(X_5) +6|X_3-1|$	$\eta _{t=0} = (2, 2, 3, 4.5, 6.5)$
	$\epsilon \sim \mathcal {N}(0,1)$, *g(1)=2, g(2)=-1, g(3)=-4*	$\eta _{t=1} = (0, 1, 2.5, 5, 7.5)$
Main features:		
*∙ * Groups	Unknown	Well-defined
*∙ * Heterogeneity	Continuous functions of covariates	Latent groups defined by covariates
*∙ * Outcome	Non-linear function	Non-linear function

**Fig. 1:**
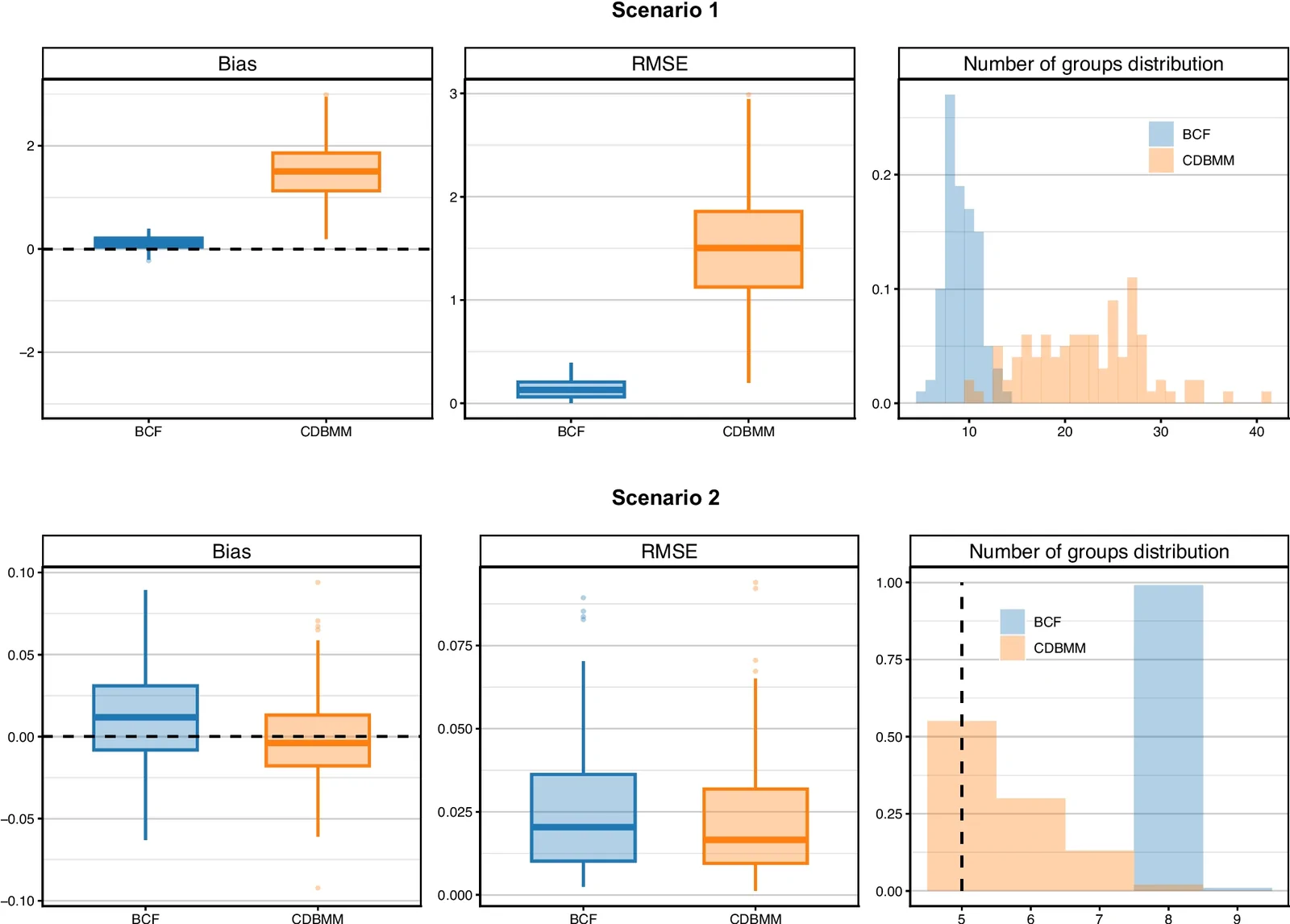
Indirect accountability simulations for BCF and CDBMM. Boxplots of bias and root mean square error (RMSE) of the average causal effect, and histograms of the number of estimated clusters estimated across 100 replicates for the two simulated scenarios. The dashed horizontal line in the bias panel indicates the target value. The dashed vertical line in the histogram indicates the true number of clusters in Scenario 2

Figure 1 compares the methods in terms of bias and root mean square error (RMSE) for the average causal effect, as well as the number of estimated clusters, across 100 replicates with sample size *n=500*. The flexibility of BCF+CART leads to strong performance in terms of bias and RMSE in both scenarios. However, the CART post-processing tends to select a similar number of clusters across settings, with a prominent mode around eight clusters, even when the underlying data-generating mechanisms differ substantially (from no clustering in Scenario 1 to a clearer five-cluster structure in Scenario 2). In contrast, CDBMM shows sensitivity to whether the data contain an underlying clustered structure: it performs poorly in Scenario 1 (where clustering is absent) but achieves good bias and RMSE in Scenario 2. Moreover, in Scenario 2, the CDBMM-induced partition concentrates around the true number of clusters, whereas the larger number of clusters estimated in Scenario 1 indicates the absence of a well-defined cluster partition.

**Fig. 2:**
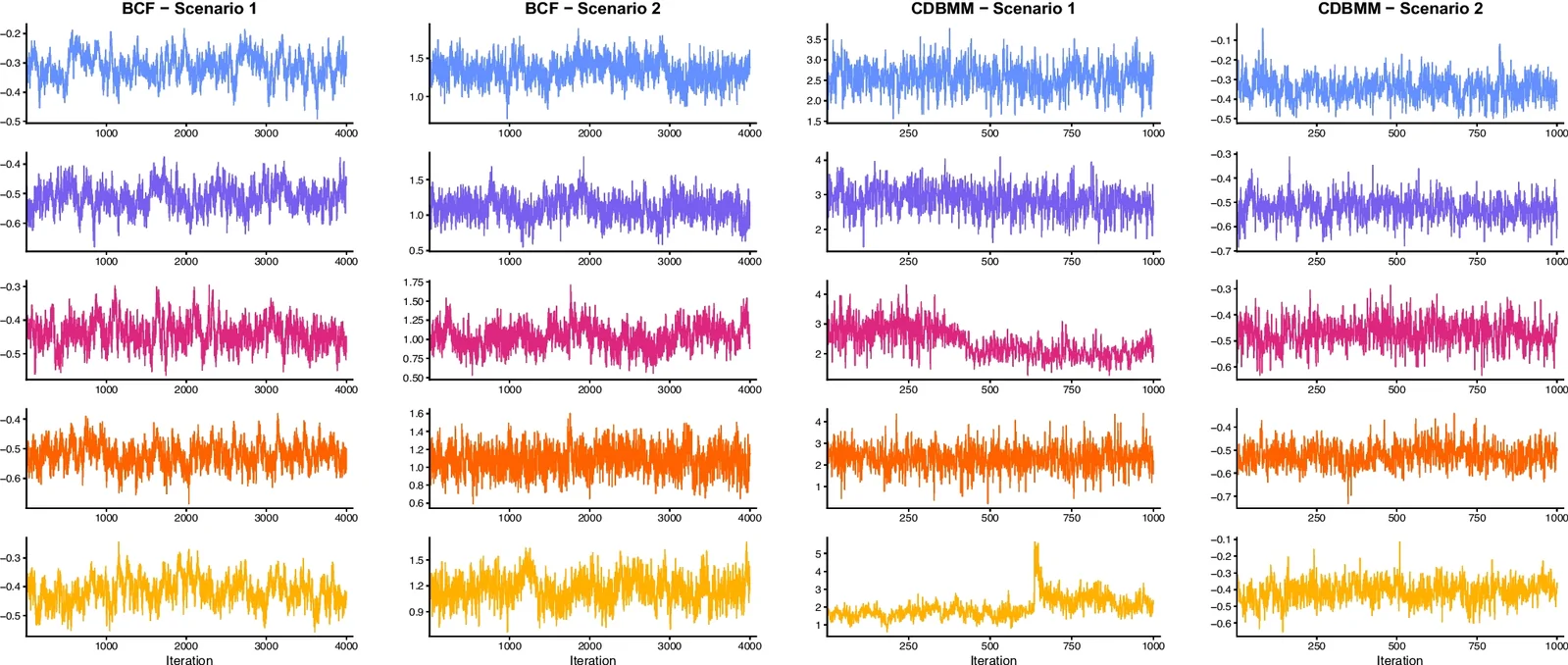
Analysis of the BCF and CDBMM convergence diagnostics: the posterior draws after burn-in of the Average Treatment Effect (ATE) for the first 5 replicates in Scenario 1 and Scenario 2. A desirable MCMC convergence pattern shows a chain that stabilizes around a constant level, with random fluctuations around a stable mean

The convergence diagnostics of the MCMC are an important aspect in the estimation of Bayesian models, particularly for complex models such as BNP approaches. Due to the high number of parameters in DDP models and the differing tree structures at each iteration in tree-based models, convergence is assessed by examining the estimation of the causal effect, in particular the ATE. Figure 2 reports the MCMC chains of the posterior draws of the ATE in the first five replicates of the two simulated scenarios for BCF and CDBMM. A desirable MCMC convergence pattern should show chains that stabilize around a constant level, with random fluctuations around a stable mean. This is achieved for almost all chains. The estimation of the third replicate of the CDBMM in Scenario 1 shows a small shift in the mean around iteration 450, while replicate five for the same setting exhibits a peak around iteration 650. This indicates that when the heterogeneous causal effect is particularly complex, it may be preferable to run the CDBMM algorithm for more iterations and increase the burn-in period.

## Direct Accountability: Evaluating Specific Interventions

Direct accountability studies evaluate whether specific environmental policies (e.g., nonattainment designations, control technology installations) lead to measurable health improvements. The main challenge lies in disentangling health effects attributable to the policy-induced pollution-reduction pathway from effects operating through other channels or from heterogeneous implementation and compliance.

As discussed in Section 2, from the potential outcomes perspective [88], this setting can be formalized within the principal stratification framework [67]. This framework presents two main challenges. First, the principal strata are not directly observable—that is, they are latent—and must be estimated. Second, both the post-intervention variables and the final outcomes involve missing counterfactual values. Consequently, it is crucial to specify a model that appropriately captures the causal relationships among these variables while allowing appropriate uncertainty propagation from the post-intervention variable model to the final outcome model.

As in indirect accountability, BNP methods have recently been applied in the principal stratification framework. Here, we compare two approaches: the Confounders-Aware SHared atoms BAyesian mixture (CASBAH) [22], which uses a DDP prior, and the Bayesian Tree-Based Principal Stratification (BPCF) [23] which employs BART priors. Additional relevant works include Schwartz, Li and Mealli [71], who proposed an infinite mixture of regression models in a principal stratification setting, and Antonelli et al. [73], who used Gaussian processes [89] to model post-intervention variables under continuous exposures.

A common feature of Bayesian approaches to principal stratification is the use of the de Finetti’s representation theorem: it is assumed that the joint distribution of the variables of interest can be exchanged between units. Specifically, the joint distribution can be written as:

$$\begin{aligned} \text {Pr}(Y(0), Y(1), P(0), P(1),T, \textbf{X})=\int _\Theta \prod _{i=1}^n \text {Pr}(Y_i(0), Y_i(1), P_i(0), P_i(1),T_i, \textbf{X}_i \mid \theta ) \text {Pr}(\theta ) d\theta , \end{aligned}$$

where $\text {Pr}(\theta )$ is the prior distribution in the parameter space *Θ * and the inner probability admits the following factorization:

$$\begin{aligned}\begin{gathered} \text {Pr}( T_i \mid \textbf{X}_i, \theta ) \cdot \text {Pr}(Y_i(0), Y_i(1) \mid P_i(0), P_i(1), \textbf{X}_i, \theta ) \cdot \text {Pr}(P_i(0), P_i(1) \mid \textbf{X}_i, \theta ) \cdot \text {Pr}( \textbf{X}_i \mid \theta ), \end{gathered}\end{aligned}$$

where the simplification on the conditional distribution of the intervention assignment follows from the assumption of strongly ignorable intervention assignment. The conditional probabilities for the post-intervention variable $\text {Pr}(P_i(0), P_i(1) \mid \textbf{X}_i, \theta )$ and for the final outcome $\text {Pr}(Y_i(0), Y_i(1) \mid P_i(0), P_i(1), \textbf{X}_i, \theta )$ are the focus of the model definition.

### CASBAH: Shared-Atoms Mixture Approach

CASBAH [22] specifies a nonparametric prior for the joint model of potential post-intervention outcomes under the two intervention levels. This enables information sharing between intervention-specific marginal distributions, while allowing confounders to inform the intervention-dependent sequences of the mixture weights. This flexible modeling framework is designed to address key challenges in principal stratification. In particular, CASBAH (i) infers the principal strata without relying on ad hoc or subjective threshold choices and (ii) provides a comprehensive uncertainty quantification for stratum membership.

The conditional model for the post-intervention variable is defined as follows:

$$\begin{aligned} P_{i} | \textbf{x}_i,t, \eta ,\sigma \sim \sum _{l \ge 1} \pi _{l}^{(t)}(\textbf{x}_i)\mathcal {N} (\eta _{l},\sigma _{l}^2), \end{aligned}$$

where the infinite sequence of weights $\left( \pi _{l}^{(t)}(\textbf{x}_i)\right) _{l \ge 1}$ is independent between the intervention level $t\in \{0,1\}$ and follows the definition of the probit stick-breaking process [79] with a multivariate Gaussian prior [90] for its parameters:

$$\begin{aligned} \pi _{l}^{(t)}(\textbf{x}_i) = \Phi (\alpha _{l}^{(t)}(\textbf{x}_i))\prod _{r<l} \left[ 1- \Phi (\alpha _{r}^{(t)}(\textbf{x}_i))\right] , \quad \alpha _{l}^{(t)}(x_i) \sim \mathcal {N}( \textbf{x}_i^T\beta _{l}^{(t)},1), \quad \beta ^{(t)} \sim \mathcal {N}_{(p+1)(L-1)}(\xi ,\Omega ). \end{aligned}$$

Information is shared across intervention levels through the common (intervention-invariant) sequences of Gaussian kernel parameters $\left( \eta _{l} \right) _{l \ge 1}$ and $\left( \sigma _{l} \right) _{l \ge 1}$. Such definition allows each unit to have a non null probability a priori to belong to the dissociative stratum, and a simple procedure to estimate the posterior probability for each unit to belong to the three strata. In particular, the posterior probability of belonging to the dissociative stratum corresponds to the proportion of times the algorithm assigns the two potential post-intervention variables, $P_i(0)$ and $P_i(1)$, to the same cluster.

The default priors and hyperprior for the Gibbs sampler implemented in PSBayes includes:

$$\begin{aligned} \eta _l {\mathop {\sim }\limits ^{iid}} \mathcal {N}(0,20), \; \sigma _l^2 {\mathop {\sim }\limits ^{iid}} \text{ InvGamma }(2,0.5), \text{ and } \beta ^{(t)} \sim \mathcal {N}_{(p+1)(L-1)}(-0.5,diag(20)), \end{aligned}$$

where *L* is the maximum number of clusters investigated by the algorithm and chosen by the user, and *diad*(20) denotes a *(p+1)(L-1)× (p+1)(L-1)* diagonal matrix with zero off-diagonal elements and diagonal entries equal to 20.

Finally, CASBAH specifies a Bayesian linear regression model for the conditional probabilities of the final outcome

$$\begin{aligned} \begin{bmatrix} Y_i(0) \\ Y_i(1) \end{bmatrix} \sim \mathcal {N}_2 \begin{pmatrix} \begin{bmatrix} \theta _{00} + \theta _{01}P_i(0) \\ \theta _{10} + \theta _{11}P_i(1)+ \theta _{12}P_i(0) + \theta _{13}P(0)P_i(1) \end{bmatrix}, \begin{bmatrix} e^{\lambda _0} & 0 \\ 0 & e^{\lambda _0 +\lambda _1 P_i(1)} \end{bmatrix} \end{pmatrix}, \end{aligned}$$

with noninformative prior distributions $\theta _l {\mathop {\sim }\limits ^{iid}} \mathcal {N}(0,20)$ and $\lambda _t {\mathop {\sim }\limits ^{iid}} \mathcal {N}(0,2)$.

### BPCF: Tree-Based Principal Stratification

Kim and Zigler [23] propose BPCF, which use two BART-based models: one for the principal stratum membership and one for the outcome, conditional on the principal strata.

Analogously to the BCF specification, the conditional probability for the post-intervention variable follows

$$\begin{aligned} P_{i} | \textbf{x}_i,t \sim \mu _P(\textbf{x}_i, \hat{\pi }(\textbf{x}_i)) + t \cdot \tau _P (\textbf{x}_i) + \epsilon _i; \end{aligned}$$

and similarly, the model for the conditional probabilities of the final outcome is

$$\begin{aligned} Y_{i} | P_{i}(0)=p_{0i}, P_{i}(1)=p_{1i}, \textbf{x}_i,t \sim \mu _Y(\textbf{x}_i, \hat{\pi }(\textbf{x}_i)) + t \cdot \tau _Y (\textbf{x}_i, p_{0i}, p_{1i}) + \epsilon '_i. \end{aligned}$$

The error terms $\epsilon _i$ and $\epsilon '_i$ are, respectively, normally distributed with local parameter 0 and scale parameter $\sigma _p$ and $\sigma _y$. The quantity $\hat{\pi }(\textbf{x}_i)$ is an estimated propensity score, and the function $\mu _P(\cdot )$ $\tau _P(\cdot )$, $\mu _Y(\cdot )$ and $\tau _Y(\cdot )$ are given independent BART priors.

BPCF provides a flexible modeling framework for both post-intervention and outcome variables. It allows heterogeneous effect on the final outcome to vary across the surface defined by continuously-scaled potential post-intervention variable, while adjusting for targeted selection and mitigating regularization-induced confounding through the inclusion of the propensity score.

Unlike CASBAH, principal strata are not inferred directly as part of the BPCF model and must be defined via a user-chosen rule (e.g., quantiles of *P(1)-P(0)* or pre-specified threshold values). Sensitivity analyses with respect to these choices are therefore strongly recommended.

### Simulation Study

To examine the finite sample behavior of the two methods, we conduct a simulation study based on the second data-generating scenarios proposed in the two previously described works [22, 23]. The principal strata are defined differently across the scenarios. In Scenario 1, ad hoc thresholds are used to partition the observations into three equally sized groups (tertiles) based on the value of $P_i(1) - P_i(0)$. In Scenario 2, units are generated directly from three principal strata, and the outcome variable is specified as a function of stratum membership. Both Scenarios induce generating processes for the outcome that complexly depend on the post-intervention and a set of confounding variables that simultaneously affect both intervention and outcome. See Table 2 for details. Both algorithms are implemented in the R software environment and are publicly available on GitHub as PSBayes and BPCF.

**Table 2:** Parameters and distributions for the simulation scenarios under direct accountability

	Scenario 1	Scenario 2
Source	Scenario 2 in Kim and Zigler (2025)[23]	Scenario 2 in Zorzetto et al (2024)[22]
Covariates	$X_{1:2} \sim \text {Unif}(0,1), X_3 \sim \mathcal {N}(1,1),$	$X_j \sim \text {Be}(\pi _j) \; j=1, 2$
	$X_{4} \sim \mathcal {N}(-1,2), X_5 \sim \mathcal {N}(1,0.5)$	*π = (0.7, 0.6)*
Intervention	$T\sim \text {Be}(f_3(X_{1:5}))$	$T\sim \text {Be}(f_4(X_{1:2}))$
	$f_3 = 0.8\Phi (0.5\mu _Y / (0.1(2-X_1-X_2)+0.25))+$	$f_4 = \text {logit}(0.4X_1+0.3X_2)$
	$$+0.1(X_1+X_2))$$	
Strata	No strata	$$S_i \in {\left\{ \begin{array}{ll} -1 & \text {if } X_{i1}=1, X_{i2}=1; \\ 0 & \text {if } X_{i1}=0; \\ 1 & \text {if } \text {otherwise}. \end{array}\right. }$$
Post-interv.	$P \sim \mathcal {N}(1.5+\mu _P(X_{1:5},T),0.1^2)$	$P_i(t) \sim \sum _{s} I_{(S_i = s)}\mathcal {N}(\eta _{t,s}, 0.05)$
	$\mu _P=0.9T +X_1 +1.5X_2+|X_3+1|+TX_4-0.5X_5$	$\eta _{t=0}= 2, \eta _{t=1}= (1,2,3)$
Outcome	$Y \sim \mathcal {N}(\mu _Y(X_{1:5})-1.5T(P(1)-P(0)) +1.5X_3,0.3^2)$	$Y(0) \sim \mathcal {N}(1+2P(0),e^{-0.5})$
	$\mu _Y = -1 +X_1-X_2+X_3+X_4+X_5$	$Y(1) \sim \mathcal {N}(1-P(0)+1.2P(1)+P(0)P(1),$
		$$e^{-0.5+0.1P(1)})$$
Main features:		
*∙ * Intervention	Non-linear function	Linear function
*∙ * Strata	Unknown	Well-defined
*∙ * Post-interv.	Non-linear function	Non-linear function
*∙ * Causal effect	Linear function	Non-linear function

**Fig. 3:**
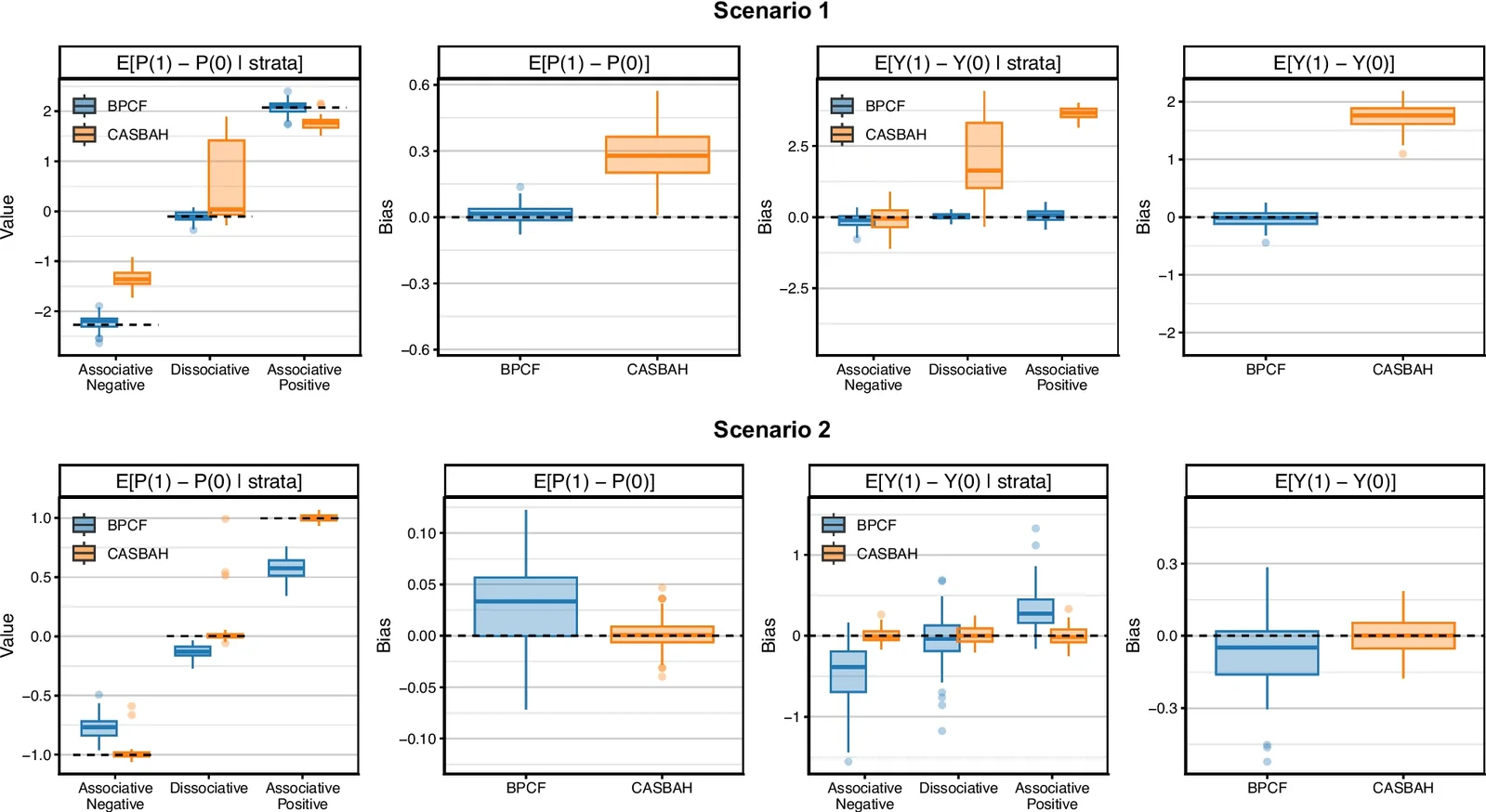
Direct accountability simulations for BPCF and CASBAH. From left to right, the plots report the values of variation of post-intervention for the three strata, the bias of $\textbf{E}[P(1)-P(0)]$, the bias for the principal causal effects for each strata, and the bias of $\textbf{E}[Y(1)-Y(0)]$. The horizontal dashed lines indicate the target values

Figure 3 illustrates the performance of the two models over 100 repetitions, each with sample size *n = 500*. We evaluate (i) stratum identification, (ii) bias in the average causal effect on the post-intervention variable, (iii) bias in the principal causal effect for the three strata, and (iv) bias in the average causal effect on the final outcome.

For the BPCF model, the strata partition is defined by the tertiles of the estimated *P(1)-P(0)*, which coincides with the data-generating mechanism in Scenario 1. On the other hand, in the CASBAH model, the point estimation of the strata partition is obtained as the output of the model algorithm. In both scenarios, model misspecification induces shrinkage of the associative strata toward zero, which in turn leads to biased estimates of the principal causal effects. Moreover, in Scenario 1, where both the post-intervention variable and the final outcome are generated from a nonlinear data-generating process, CASBAH yields biased estimates, likely due to the linear specification of the outcome model. In contrast, BPCF performs poorly in Scenario 2, where the principal stratum membership is clearly defined.

**Fig. 4:**
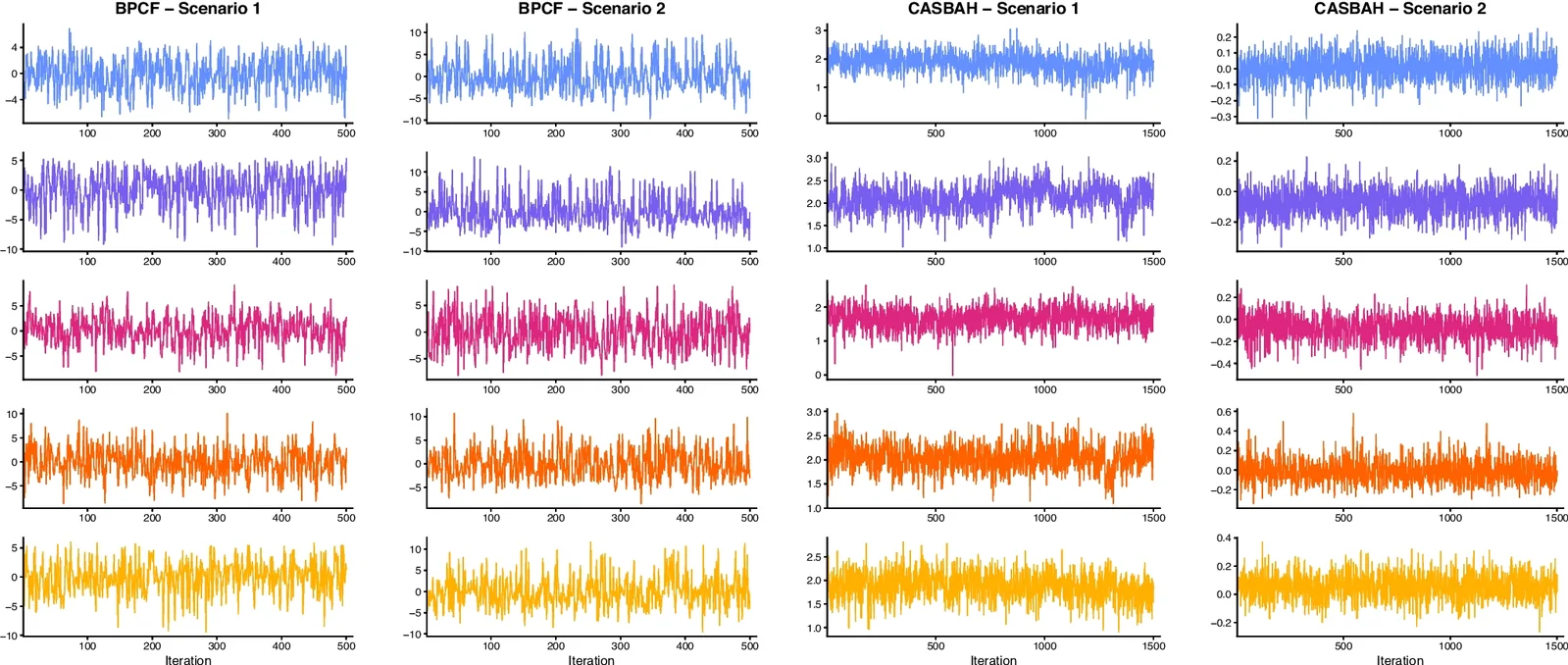
Analysis of the BPCF and CASBAH convergence diagnostics: the posterior draws after burn-in of the Average Treatment Effect (ATE) for the first 5 replicates in Scenario 1 and Scenario 2. A desirable MCMC convergence pattern shows a chain that stabilizes around a constant level, with random fluctuations around a stable mean

We analyze the convergence of the two models by examining the posterior draws of the ATE in the first five replicates of the two simulated scenarios. Figure 4 shows MCMC chains after burn-in that stabilize around a constant level, with random fluctuations around it, as expected under good convergence diagnostics. The different ranges of fluctuation reflect the variability of the model estimates, and, in accordance with Fig. 3, we can see that BPCF exhibits a wider range than CASBAH. Similarly, the average values of the MCMC chains represent the estimated ATE, showing good performance except for CSBAH in scenario 1, which is biased. Across different replicates, both models report similar results, confirming the robustness of the two approaches.

## Discussion and Comparison

### Summary Comparison

Table 3 highlights how the four BNP approaches considered in this review map naturally to the two accountability paradigms. BCF+CART and CDBMM are designed for *indirect accountability*, where the primary goal is to learn an exposure-effect relationship and characterize heterogeneity in the ensuing causal effects. In contrast, CASBAH and BPCF address *direct accountability*, where inference is complicated by policy implementation and compliance, and where principal stratification offers a principled way to separate effects among units whose exposures respond differently to the intervention.

**Table 3:** Summary comparison of four BNP methods

Feature	BCF+CART	CDBMM	CASBAH	BPCF
Research Question				
Direct accountability	✗	✗	✓	✓
Indirect accountability	✓	✓	✗	✗
Approach				
Tree-based	✓	✗	✗	✓
Mixture-based	✗	✓	✓	✗
Group Discovery				
Native	✗	✓	✓	✗
Post-hoc	✓	✗	✗	✓
Uncertainty				
For groups	✗	✓	✓	✓
For effects	✓	✓	✓	✓
Software	bcf, rpart	HTEBayes	PSBayes	BPCF

A second organizing axis is the modeling strategy. BCF+CART and BPCF rely on tree-based priors (BART) to flexibly learn nonlinearities and interactions, often with strong empirical performance and relatively minimal tuning. CDBMM and CASBAH instead exploit the partition-inducing nature of Bayesian mixtures: clustering emerges as a byproduct of the model, and inference can be reported at both the unit and subgroup levels. These differences matter because accountability questions are often communicated in terms of *which communities* experience larger benefits. Mixture models provide a native notion of subgroup discovery with posterior uncertainty on the partition, whereas tree-based approaches often require an additional summarization step, such as post-hoc CART, to produce interpretable groups.

The role of group discovery is therefore central. In BCF+CART, subgrouping is explicitly a second-stage procedure: the causal model produces posterior draws (or point estimates) of heterogeneous effects, and CART is used to summarize them. This workflow is simple and practical, but uncertainty quantification for the groups defined by the CART tree is not automatically available and typically requires additional steps. In contrast, CDBMM and CASBAH induce partitions within the Bayesian model, identify groups, and principal strata respectively. This yields coherent posterior uncertainty on both (i) the effect estimates within groups/strata and (ii) the grouping itself. BPCF provides posterior uncertainty for effects, and it can support group summaries (e.g., via posterior summaries of effects over covariate-defined regions or by stratifying based on estimated principal effects), but the principal strata definition is not a native object since it relies on ad hoc rules specified by the user, as opposed to mixture models where group discovery is built-in.

Finally, Table 3 emphasizes practical considerations. All four methods provide posterior uncertainty for causal effects, but their computational profiles differ. BART-based methods often scale well to moderately large datasets and are supported by mature software. Mixture-based approaches can be more computationally demanding because they must explore partitions and mixture weights, but they repay this cost by producing interpretable latent subgroup structure with coherent uncertainty quantification. In practice, the choice is often driven by a combination of the scientific estimand (indirect vs. direct accountability), the need for explicit group discovery, and the demands of computation and interpretability.

### When to Use Which Method

A useful decision rule is to begin with the accountability question and then consider the role of subgroup discovery.

#### Indirect Accountability.

If the goal is to estimate a flexible exposure-effect relationship and characterize heterogeneity across observed covariates, BCF is a strong default. Its decomposition into prognostic and treatment-effect components and the inclusion of the propensity score help mitigate regularization-induced confounding, a common concern in flexible regression models for causal inference. The BCF+CART extension is particularly attractive when the primary deliverable is an interpretable set of subgroups and corresponding GATE summaries. However, practitioners should recognize that CART is a descriptive, second-stage tool: unless additional steps are taken, it does not deliver fully Bayesian uncertainty quantification for the selected partition.

CDBMM is most attractive when subgroup discovery is a central scientific objective and when the practitioner seeks posterior uncertainty on the partition. In settings where the data plausibly arises from distinct subpopulations with different vulnerability profiles—e.g., when vulnerability is expected to concentrate in a limited set of communities defined by correlated socioeconomic and health characteristics—mixture-based modeling can provide a compelling representation. The simulation results in Section 3 also highlight an important practical consideration: mixture-based methods are sensitive to whether clustering is truly present. When there is no meaningful cluster structure, CDBMM may overfit by inducing spurious partitions that do not translate into improved causal effect estimation. In such settings, BCF may provide more stable performance; however, it does not yield a native clustering structure.

#### Direct Accountability.

When evaluating a specific policy intervention, principal stratification becomes valuable because interventions often affect exposures unevenly across geographical locations or populations. CASBAH is particularly appealing when principal strata are conceptually meaningful but difficult to define by thresholds. Its shared-atoms construction provides a data-adaptive approach to identifying strata and quantifying uncertainty about membership, avoiding ad hoc cutoffs on the post-intervention contrast. This is especially useful in regulatory settings where the pollution response may be continuous and noisy, making threshold-based compliance definitions unstable. Furthermore, CASBAH provides a probabilistic definition of principal stratum membership, which, in this context, translates into a probabilistic representation of non-compliance. This is useful to understand the differential uptake of the environmental policy itself.

BPCF is attractive when one expects complex nonlinear relationships among covariates, pollution changes, and health outcomes, and when the scientific question concerns smooth effect variation over a continuous intermediate. Its tree-based structure can flexibly model both the post-intervention process and the outcome, and it inherits the regularization properties of BART. At the same time, BPCF typically requires the practitioner to define strata or effect summaries using a chosen function of (*P*(1), *P*(0)) (e.g., quantiles or thresholds). This flexibility is useful but introduces an additional layer of practitioner decisions, making sensitivity analyses essential.

### Key Contributions

Taken together, these four BNP approaches advance the accountability literature in several ways.

First, they provide principled, data-adaptive characterizations of heterogeneity. Rather than relying on a limited menu of pre-specified subgroups, they allow effect variation to be discovered from the data, in a data-adaptive manner, while borrowing strength across similar observations. This is particularly important in environmental health, where vulnerability may emerge through complex interactions among socioeconomic status, baseline health, co-pollutant mixtures, and geography.

Second, they reduce reliance on arbitrary thresholds. This contribution is especially salient for direct accountability, where strata definitions based on cutoffs in pollution change are common but often scientifically and statistically fragile. CASBAH offers a clear alternative by treating strata as latent and inferring them with uncertainty. Even under indirect accountability, mixture-based group discovery (CDBMM) provides an alternative to hard-coded subgroup analyses.

Third, all four approaches deliver posterior uncertainty quantification. Accountability studies are frequently used to inform policy decisions, and quantifying uncertainty is essential for transparent interpretation. BNP methods naturally propagate uncertainty from the outcome model to estimands such as CATEs, GATEs, and principal effects, and they allow uncertainty statements to be made at the level of covariate-defined targets.

Fourth, the methods clarify the match between estimand and design. Indirect accountability is fundamentally about the estimation of exposure-effect relationships, whereas direct accountability is about the causal effect of a specific intervention operating through heterogeneous implementation and compliance. Treating these as distinct inferential problems—rather than variants of the same regression task—is one of the most important conceptual contributions of the accountability framework, and the methods reviewed here provide concrete statistical tools aligned with that distinction.

### Limitations

Despite their advantages, BNP methods also present limitations that matter in applied accountability research.

#### Computation and Scaling.

All four approaches can be computationally intensive, particularly for large administrative datasets or national-scale spatiotemporal studies. While BART-based methods often scale reasonably well, mixture-based methods may require substantial computation to explore partitions and mixture weights. For very large datasets, practitioners may need to consider strategies such as subsampling, minibatching, or modeling on aggregated units (e.g., county-year summaries) when scientifically defensible.

#### Covariate Selection and Causal Identification.

  As emphasized in Section 2, no flexible method can compensate for poor identification. Each of the models introduced still rely on causal identifying assumptions such as SUTVA and strong ignorability (for indirect accountability) or principal ignorability and monotonicity (for direct accountability). In practice, careful and expert-based discussions of each assumption remains essential. In this sense, of particular importance is the definition of the covariates to be included in the study. Including strong confounders is critical, but including variables that induce post-intervention bias (in indirect accountability) or collider bias can be harmful. Moreover, positivity (overlap) constraints may be more binding in flexible models that target fine-grained heterogeneity, motivating diagnostics and, when needed, restrictions to regions of covariate overlap.

#### Interpretability and Communication.

A recurring challenge in environmental health is communicating complex heterogeneity results to policymakers and stakeholders. While BNP methods can provide rich descriptions of effect variation, reporting must remain policy-relevant and interpretable. Group-level summaries (e.g., GATEs) and carefully chosen visualizations are often more actionable than high-dimensional surfaces. Mixture models offer a natural pathway to communicate subgroup results, but the notion of a latent cluster can still be difficult to interpret, especially when clusters are defined by many correlated covariates.

#### Defining Heterogeneity: Individuals vs Groups.

A critical interpretive limitation concerns the level at which heterogeneous treatment effects are defined [91]. Individual treatment effects $\tau _i = Y_i(1) - Y_i(0)$ require the joint distribution of potential outcomes and involve fundamentally non-identifiable “cross-world” dependence: even with infinite data, one cannot learn whether a particular individual would have benefited from treatment (read, exposure or intervention). The BNP methods reviewed here instead target *conditional average treatment effects* defined over covariate regions. However, when the covariate space is high-dimensional, it can be difficult to investigate causal effects across the entire space, and *group average treatment effects* can provide a useful and interpretable summary. The identified groups by the proposed BNP models are characterized by units that have similar causal effects within the group and different effects across groups. Finally, although continuous-exposure frameworks for estimating exposure-response curves exist [35] and have been applied in environmental epidemiology [36], none of these methods characterizes treatment effect heterogeneity in a data-driven manner; instead, they require the subgroups of interest to be pre-specified ex ante (for a discussion of the limitations of pre-specified HTE analysis, see Athey and Imbens [15]).

Understanding the characteristics of the groups is essential to inform policy decisions. These methods, if carefully implemented under a strong causal framework, have the potential to inform *targeted interventions* or *treatment rules* based on observable characteristics (e.g., prioritizing pollution controls in communities with high estimated CATEs), but they cannot identify which specific individuals will respond. The posterior distribution of *τ (x)* quantifies uncertainty about population-level heterogeneity and the mapping from covariates to effects, not uncertainty about individual counterfactual responses.

## Future Directions

Several directions appear especially promising for extending the BNP accountability methods. First, scalability and computation remain active areas. Faster posterior inference, approximate Bayesian computation for BNP models, and hardware-aware implementations could broaden the applicability of these tools to national administrative databases and high-resolution exposure surfaces. Second, integrating interference and spillovers into accountability designs is increasingly important [92, 93]. Air pollution and regulatory actions often involve spatial and economic spillovers, potentially violating SUTVA. Extending BNP causal methods to settings with interference, while retaining interpretable heterogeneity summaries, would strengthen causal claims in many real-world applications. Third, bridging indirect and direct accountability remains a key goal. Indirect studies estimate exposure effects, while direct studies evaluate specific interventions that alter exposures imperfectly. Methods that combine these perspectives, for example, by jointly modeling policy-induced exposure changes and outcome responses, or by transporting causal estimates across contexts, could provide more comprehensive evidence for regulatory impact assessments.

### Methodological Extensions

A natural next step is to move beyond binary contrasts and develop BNP methods for *continuous exposures* and full *exposure-response surfaces* [35, 94, 95]. Many accountability questions concern marginal exposure reductions and nonlinearity in the exposure-response relationship, which cannot be fully captured by dichotomization. BNP models that learn smooth, covariate-dependent dose-response functions would better align statistical estimands with policy levers that shift exposure distributions rather than toggling exposure status.

Accountability designs also increasingly require handling *time-varying exposures and interventions*. Regulations are implemented gradually, compliance evolves, and health responses may depend on exposure histories. Extending BNP causal methods to longitudinal regimes would allow investigators to estimate dynamic causal effects under sustained or phased interventions, while addressing time-varying confounding and delayed effects. This is relevant for both indirect accountability (exposure trajectories) and direct accountability (policy intensity over time).

For direct accountability in particular, many interventions operate through more than one post-intervention process. This motivates extensions to *multiple intermediates and pathways*, where pollution change is one mediator among several (e.g., co-pollutants, economic activity, health care access, monitoring intensity). BNP principal stratification could be generalized to multivariate intermediates to characterize which combinations of pathways are activated and where, providing more realistic mechanistic insight than a single-intermediate formulation.

In parallel, addressing *spatial correlation and interference* is increasingly urgent. Environmental exposures and regulatory actions produce spatial spillovers that can violate SUTVA: emissions reductions in one county can affect air quality downwind, and policy designation can shift industrial activity across boundaries. BNP methods that incorporate spatial structure and explicitly model interference—while still delivering interpretable heterogeneity summaries—would strengthen causal claims in many accountability analyses and improve the credibility of policy-relevant inference [96–99].

Finally, principal stratification and mediation analysis offer complementary lenses for direct accountability, suggesting benefits from deeper *integration with mediation analysis* [61, 63]. Principal stratification targets heterogeneity in whether and how an intervention changes intermediates (e.g., compliance), while mediation decomposes total effects into pathway-specific components. Unified BNP frameworks that jointly represent compliance heterogeneity and pathway decomposition could support richer answers to policy questions about *how* interventions work, *for whom*, and *through which channels*.

### Applications

Several applied areas are especially well suited to BNP accountability tools. One is *multi-pollutant mixtures*. Real-world exposures consist of correlated pollutant combinations whose composition varies across space and time, and policies often affect several pollutants simultaneously by targeting sources. BNP models are well positioned to represent nonlinear and interactive mixture effects and to characterize heterogeneity in those effects across communities, allowing both indirect and direct accountability analyses to move beyond single-pollutant contrasts.

A second application is *environmental protection prioritization*. Accountability studies increasingly aim to quantify not only average benefits, but also whether regulations reduce disparities and which communities remain at highest risk. BNP heterogeneity estimates can support prioritization by identifying covariate-defined vulnerability profiles and by quantifying uncertainty in subgroup comparisons that are central to equity-focused decision-making. Importantly, these applications require careful communication: heterogeneity results should be translated into policy-relevant summaries that avoid overinterpretation of individual-level “responder” claims.

A third domain is *global environmental health*. Many low- and middle-income settings face high exposures, rapidly changing regulations, and sparse monitoring. BNP methods that borrow strength across areas, accommodate measurement error, and support transportability of exposure–response relationships could facilitate accountability analyses in contexts where data are limited and policy mechanisms differ from those in high-income countries.

## Conclusion and Recommendations

For researchers and practitioners, the starting point should be to *clearly distinguish direct from indirect accountability questions*, since the estimand, identification assumptions, and modeling priorities differ based on which accountability approach is adopted. Indirect accountability centers on the effects of exposures and covariate-dependent heterogeneity; direct accountability centers on the causal effects of specific interventions, often requiring explicit consideration of post-intervention processes such as pollution change and differential implementation. Once researchers have identified the accountability research question they want to answer, strong emphasis should be given to a careful assessment of the plausibility of the underlying causal assumptions on which the causal credibility of the subsequent data analysis relies. This verification and assessment of the causal assumptions should come before any method is chosen and/or implemented. Once the research question is defined and the identifying assumptions evaluated, the method choice is the next step.

In the context of modern accountability analyses, as we have seen, emphasis is placed not only on population-level effects but also on effect heterogeneity, which allows researchers to answer questions such as: “does the harm/benefit of the exposure/intervention varies based on different communities?” In this context, tree-based approaches are often effective defaults when flexible effect surfaces and strong predictive performance are needed with relatively straightforward implementation, whereas mixture-based approaches are especially valuable when native subgroup discovery and coherent uncertainty about partitions are central scientific goals.

Across both direct and indirect accountability paradigms, researchers should prioritize *extensive sensitivity analyses*, not only for modeling choices, but also for identification assumptions (e.g., unmeasured confounding and overlap in indirect studies; principal ignorability and monotonicity in direct studies) and subgroup discovery (e.g., are subgroups stable?). Finally, because accountability evidence is used in high-stakes regulatory contexts, reproducibility is essential: sharing code, documenting preprocessing steps, and reporting diagnostics should be treated as core components of the analysis rather than optional add-ons.

For policymakers, several messages follow directly from the heterogeneity focus of BNP accountability methods. First, *heterogeneity is the rule, not the exception*: the same exposure reduction or regulation can yield different health benefits across communities because baseline health, socioeconomic conditions, co-exposures, and implementation intensity vary. Second, *different populations experience different effects*, and the communities most burdened by pollution are often also those most vulnerable; accounting for heterogeneity is therefore central to both efficiency and equity. However, *heterogeneity estimates should be interpreted with appropriate caution*: these methods assess differences in average effects across covariate-defined subgroups, not individual-level responses, and results carry posterior uncertainty, on discovered subgroups and estimated effects, that should be communicated transparently to avoid overconfident targeting of specific communities. Third, *direct evaluation of policies complements exposure effects evidence*: indirect studies inform what might happen under hypothetical exposure reductions, while direct studies provide evidence on what did happen following implementation of a particular policy in a particular setting.

Finally, *mechanistic understanding improves actionability*. Principal stratification, as reviewed here, can clarify whether health improvements occurred primarily in areas that complied with regulatory requirements and achieved actual pollution reductions. However, understanding the specific causal pathways through which policies affect health—whether through air quality improvements, changes in economic activity, healthcare access, or behavioral responses—requires mediation analysis, which was beyond the scope of this review but represents an essential complement to principal stratification in direct accountability. Together, these frameworks help decision-makers identify where implementation succeeded, where it failed, and what complementary supports might be needed to ensure health benefits are realized.

## Key References

- P. R. Hahn, J. S. Murray, and C. M. Carvalho. Bayesian regression tree models for causal inference: Regularization, confounding, and heterogeneous effects (with discussion). *Bayesian Analysis*, 15(3):965–1056, 2020.
  - ○ They introduce the BCF model to estimate the conditional average causal effect under indirect accountability.
- C. Kim and C. Zigler. Bayesian nonparametric trees for principal causal effects. *Biometrics*, 81(1):ujaf024, 2025.
  - ○ They introduce the BPCF model to estimate the principal causal effects under direct accountability.
- C. M. Zigler and F. Dominici. Clarifying Policy Evidence With Potential-Outcomes Thinking–Beyond Exposure-Response Estimation in Air Pollution Epidemiology. *American Journal of Epidemiology*, 180(12):1133–1140, 2014.
  - ○ The manuscript defines the countability framework according to the guidelines of the Health Effect Institute.
- D. Zorzetto, F. J. Bargagli-Stoffi, A. Canale, and F. Dominici. Confounder-Dependent Bayesian Mixture Model: Characterizing Heterogeneity of Causal Effects in Air Pollution Epidemiology. *Biometrics*, 80(2):ujae025, 2024.
  - ○ They introduce the CDBMM model to estimate the group average causal effect under indirect accountability.
- D. Zorzetto, A. Canale, F. Mealli, F. Dominici, and F. J. Bargagli-Stoffi. Bayesian Nonparametrics for Principal Stratification with Continuous Post-Treatment Variables. *arXiv preprint arXiv:2405.17669*, 2024.
  - ○ They introduce the CASBAH model to estimate the principal causal effects under direct accountability.

## Software

The R code implementing the methods described in Sections 3 and 4 is available in the respective GitHub repositories referenced in these publications. The code to reproduce simulation results is available at github.com/emma-landry/BNP-HTE-EnvironmentalHealth.

## Data Availability

No datasets were generated or analysed during the current study.
